# Factors modulating perception and production of speech by AI tools: a test case of Amazon Alexa and Polly

**DOI:** 10.3389/fpsyg.2025.1520111

**Published:** 2025-03-13

**Authors:** Jae Yung Song, Charles Rojas, Anne Pycha

**Affiliations:** ^1^Department of English Language and Literature, Chung-Ang University, Seoul, Republic of Korea; ^2^Department of Linguistics, University of Wisconsin-Milwaukee, Milwaukee, WI, United States

**Keywords:** artificial intelligence, speech recognition, word frequency, neighborhood density, speaking rate, adaptation

## Abstract

To develop AI tools that can communicate on par with human speakers and listeners, we need a deeper understanding of the factors that affect their perception and production of spoken language. Thus, the goal of this study was to examine to what extent two AI tools, Amazon Alexa and Polly, are impacted by factors that are known to modulate speech perception and production in humans. In particular, we examined the role of lexical (word frequency, phonological neighborhood density) and stylistic (speaking rate) factors. In the domain of perception, high-frequency words and slow speaking rate significantly improved Alexa’s recognition of words produced in real time by native speakers of American English (*n* = 21). Alexa also recognized words with low neighborhood density with greater accuracy, but only at fast speaking rates. In contrast to human listeners, Alexa showed no evidence of adaptation to the speaker over time. In the domain of production, Polly’s vowel duration and formants were unaffected by the lexical characteristics of words, unlike human speakers. Overall, these findings suggest that, despite certain patterns that humans and AI tools share, AI tools lack some of the flexibility that is the hallmark of human speech perception and production.

## 1 Introduction

Human-AI (artificial intelligence) interactions are quickly becoming an integral part of our daily lives, as witnessed by the rising use of AI tools. AI tools, such as Amazon’s Alexa and Apple’s Siri, utilize a wide range of technologies, including natural language processing, automatic speech recognition (ASR), and machine learning, to simulate human conversation (Harvill et al., 2024). One of the ultimate goals of these technologies is for AI tools to reach a level of verbal communication which mimics that of human speakers and listeners. To accomplish this goal, we must have a deeper understanding of the ways in which different factors interact with the processing and production of spoken language. While the psycholinguistics literature has identified a multitude of factors that influence the human ability to recognize and produce words, very little is known about whether these same factors affect AI tools. Thus, this study’s purpose was to examine the extent to which AI tools are impacted by the same factors that affect word recognition and production in humans.

This type of investigation is important in order for psycholinguistic researchers, and the scientific population more generally, to be informed consumers of AI. For any given factor that affects word recognition and production in humans, the speech behavior of AI tools has the potential to faithfully replicate that behavior or to distort it—and this, in turn, affects how human users will respond. For example, if an AI tool faithfully replicates human speech behavior across a wide variety of factors, we might expect human users to respond to it in ways that resemble their interactions with other humans, and potentially to anthropomorphize it. On the other hand, if AI tools distort certain factors of human speech behavior, human users may undergo an experience of uncanniness, and respond to the tool in unpredictable ways. By investigating how AI tools are impacted by psycholinguistic factors, we take an important step toward understanding how such tools affect our own behavior as human beings.

In human listeners, the accuracy of spoken word recognition depends on a combination of at least three types of factors, as suggested by Bradlow and Pisoni (1999, p. 2083): (1) lexical factors, such as word frequency and phonological neighborhood density, (2) style- or signal-related factors, such as speaking rate, and (3) instance-specific factors, such as the listener’s prior experience with the talker’s voice. These factors, which affect the recognition of words in human speakers, are also likely to affect the production of those same words. Below we will expand on how each of the three types of factors can affect the human ability to recognize and produce words.

First, as for the lexical characteristics of words, it has been well documented in the literature that high-frequency words are privileged over low-frequency words in speech perception (Todd et al., 2019). Listeners recognize high-frequency words more accurately and quickly than low-frequency words (Howes, 1957; Broadbent, 1967; Luce and Pisoni, 1998). During lexical decisions, high-frequency words are judged as real words more often and faster relative to low-frequency words (Forster and Chambers, 1973; Luce and Pisoni, 1998). In speech production, high-frequency words are produced more casually (or less clearly) than low-frequency words, with shorter vowel durations and more contracted vowel spaces (Munson and Solomon, 2004).

Phonological neighborhood density is a measure of the number of similar-sounding words that a target word has (Luce and Pisoni, 1998). Numerous studies on speech perception have demonstrated that words with many similar-sounding words (i.e., neighbors) are recognized more slowly and less accurately due to the presence of their neighbors, which act as competitors, compared with words with few similar-sounding words (Luce and Pisoni, 1998; Vitevitch and Luce, 1998; Ziegler et al., 2003; Vitevitch and Luce, 2016; Van Engen, 2017). Contrary to research on speech perception, studies on speech production have yielded inconsistent findings about the effect of phonological neighborhood density. Some studies have shown that words from dense neighborhoods are more clearly articulated, probably as a result of talkers attempting to maximize intelligibility of words that might otherwise be confused (Munson and Solomon, 2004; Watson and Munson, 2008; Scarborough and Zellou, 2013); in contrast, other studies have shown that words from dense neighborhoods are less clearly articulated, suggesting that words that are retrieved quickly are more likely to be reduced (Yao, 2011; Gahl et al., 2012).

Some studies have looked at the joint effects of word frequency and phonological neighborhood density. Their findings have shown that lexically “easy” words (i.e., frequent words with few phonological neighbors) are recognized more accurately than lexically “hard” words (i.e., infrequent words with more phonological neighbors) (Bradlow and Pisoni, 1999). In speech production, “hard” words are more carefully articulated, as indicated by longer vowel durations and expanded vowel spaces (Munson and Solomon, 2004; Wright, 2004).

Second, a large amount of evidence suggests that changes in speech style-related variables affect speech production and perception. According to Lindblom (1990)’s Hyper- and Hypo-articulation (H&H) Model, speakers constantly monitor listeners’ needs and vary their speech output along a continuum of hypo-speech (which reflects desire to minimize articulatory effort) and hyper-speech (which reflects desire to be understood better). Clear speech, which is a distinct speaking style that the speaker adopts in order to be better understood by a listener, is a good example of hyper-speech. Switching from conversational speech production to clear speech gives rise to a number of acoustic changes, including slower speaking rates and more carefully articulated vowels and consonants (Picheny et al., 1986; Bradlow, 2002; Smiljanić and Bradlow, 2005, 2009; Uther et al., 2007). Extensive research has indicated that words produced in a clear speech style are more intelligible than those produced in a casual speech style, and this has been demonstrated in various populations including hard-of-hearing adult listeners (Picheny et al., 1985), normal-hearing adult listeners (Bradlow et al., 1996; Krause and Braida, 2002; Hazan et al., 2012), nonnative adult listeners (Bradlow and Bent, 2002; Smiljanić and Bradlow, 2011), school-aged children with and without learning disabilities (Bradlow et al., 2003), and infants (Song et al., 2010). Relatedly, Bradlow and Pisoni (1999) showed that words produced at a slow and medium speaking rate were recognized significantly more accurately than words produced at a fast rate. However, they found no intelligibility advantage for the slow speaking rate over the medium speaking rate.

Third, studies have demonstrated that word recognition accuracy is enhanced when the listener is familiar with the talker’s voice. For example, Nygaard et al. (1994) showed that listeners were better at identifying novel words in noise when the words were produced by a talker they had previously been trained to identify than when the same words were produced by a novel talker. Thus, learning the specific acoustic properties of a talker’s voice seemed to facilitate the subsequent phonetic analysis of spoken words. Similarly, Bradlow and Pisoni (1999) showed that hard words presented later in a session were recognized more accurately than hard words presented earlier in the session. In contrast, easy words presented later in the session and those presented earlier in the session did not significantly differ in intelligibility. Overall, the results suggested that the intelligibility challenge introduced by the lexical characteristics of hard words may be overcome as the listener becomes accustomed to the talker’s voice.

Compared to the wealth of psycholinguistics literature on the factors influencing human word recognition and production, only a handful of studies have examined whether these same factors affect machine recognition and production systems. Earlier research on then state-of-the-art ASR systems showed that their recognition errors increased for very slow or fast speech (Siegler and Stern, 1995; Fosler-Lussier and Morgan, 1999; Greenberg and Chang, 2000; Hirschberg et al., 2004) and infrequent words (Fosler-Lussier and Morgan, 1999; Mansfield, 2021). To the best of our knowledge, there have been only two studies that examined the role of neighborhood density in ASR performance. Goldwater et al. (2010) analyzed the effects of a wide range of prosodic, lexical, contextual, and disfluency factors on the accuracy of two ASR systems. They identified several factors associated with high error rates, including disfluencies, closed-class words, short words, and words at the beginning of a turn. In addition, they found no correlation between the number of neighbors and error rates of words presented in context, suggesting that phonological neighborhood density might play a more significant role when contextual cues are insufficient to distinguish words that sound similar. In contrast, Jyothi and Livescu (2014) showed that their novel relative frequency-weighted measures of word neighborhood effectively predicted errors from both isolated-word and continuous-word ASR systems.

The process of evaluating the ASR systems in these studies typically involved comparing system transcriptions of conversational speech data with reference transcriptions generated manually by humans. Thus, all of the aforementioned studies used the existing datasets of conversational speech, such as the Switchboard Corpus, to evaluate ASR systems. However, recent advancements in technology have made it feasible to examine real-time conversations between humans and voice-activated AI assistants. This presents an opportunity to gain further insight into the factors influencing the accuracy of spoken word recognition during real-time interaction.

Recent years have seen several exciting lines of new questions about AI tools emerge from psycholinguistic research, including: What speech adjustments do speakers make when speaking to a voice-activated AI assistant as opposed to a human (Cohn and Zellou, 2021; Cohn et al., 2021, 2022, 2024)? How can the use of AI tools facilitate the acquisition of a second language (Dizon, 2017, 2020; Moussalli and Cardoso, 2020; Song et al., 2022; Chen et al., 2023; Hsu et al., 2023; Tai and Chen, 2023; Luo et al., 2024; Tai, 2024; Wang et al., 2024; Yang et al., 2024)? How do people adopt or respond to the various attributes of AI-generated voice (Schreibelmayr and Mara, 2022; Dodd et al., 2023; Gwizdzinski et al., 2023; Zellou et al., 2023; Pycha and Zellou, 2024)? Despite the growing interest in AI tools, the factors accounting for the recognition and production patterns of AI tools have not been well understood.

Thus, the primary goal of this study was to examine whether the factors that affect word recognition and production in humans are also shared by AI tools. We tested two AI tools developed by Amazon: their voice-activated AI assistant, Alexa, and their text-to-speech AI service, Polly. In Experiment 1, we looked at how Alexa’s word recognition was affected by two lexical factors (word frequency and phonological neighborhood density) and a style-related factor (speaking rate). In addition, we examined how an instance-specific factor (the listener’s prior experience with the talker’s voice) influenced Alexa’s word recognition. In Experiment 2, we examined how the acoustic properties of speech generated by Polly changed as a function of the lexical properties of words (word frequency and phonological neighborhood density) and style (speaking rate).

Note that, although both Alexa and Polly make use of AI technology, these two tools serve different purposes. Alexa is an interactive assistant, designed to interact with people in a natural manner: an individual can query Alexa by speaking, and receive a spoken response. Polly, on the other hand, is a text-to-speech (TTS) tool: an individual can type a sentence or paragraph, and Polly will speak it aloud. Ideally, we might have tested a single tool, such as Alexa, on both perception and production. In practice, however, this was not feasible, because it is not possible to elicit a pre-defined spoken stimulus from Alexa. In the current study, therefore, Experiment 1 tests the ASR component of Alexa, while Experiment 2 tests Polly, which is a TTS tool. Note also that, because Polly is a TTS tool, it produces speech output from text alone. Therefore, it is not possible to examine how prior experience with a talker’s voice would affect its behavior. For this reason, Experiment 2 only examines lexical and style factors, and not an instance-related factor.

Studies comparing human and machine recognition systems have long indicated that ASR performance is not up to par with human performance, especially when dealing with degraded signals and highly variable spontaneous speech (Lippmann, 1997; Dusan and Rabiner, 2005; Juneja, 2012). Despite the significant advancement made over the last decade in ASR, with recognition rates approaching human levels (Xiong et al., 2016; Hussein et al., 2022), ASR performances still lag behind in several areas. One area where human and machine recognition systems diverge greatly is perceptual adaptation. For instance, human listeners show improvements in the speed and accuracy of word identification after only brief exposure to unfamiliar accented speech, suggesting rapid perceptual adaptation (Clarke and Garrett, 2004; Adank and Janse, 2010; Xie et al., 2018). Although there is evidence that modern ASR systems can adjust their internal processes to mimic human perceptual adaptation (Winata et al., 2020), it is still unclear whether these systems can exhibit rapid learning to a similar degree as humans (Davis and Scharenborg, 2016).

Similar limitations are found in speech produced by AI tools based on neural networks. Alexa and Polly both progressed from the earlier unit-selection method of stringing together small snippets of pre-recorded sounds to neural-network-based text-to-speech systems, which synthesize speech from scratch. Compared to speech produced by unit selection, speech produced by neural text-to-speech systems is generally considered to be more natural-sounding and more versatile (Trueba and Klimkov, 2019). Nonetheless, there is evidence that current neural text-to-speech systems often fail to capture sophisticated linguistic information that underlies speech. For example, Fernández-Peña (2023) demonstrated that Polly produced intonation patterns that do not match the pragmatic meanings of tag questions.

Based on the previous research, in Experiment 1, we predicted that lexical- and style-related factors would influence Alexa’s recognition errors in a similar way to humans, with higher error rates for low frequency words, high density words, and fast speech. In contrast, we predicted that Alexa’s recognition errors would not necessarily decrease with increased experience with the speaker’s voice, indicating a lack of human-like perceptual flexibility and rapid learning. In Experiment 2, we predicted that Polly’s speech production would be modulated by speaking rate, but not necessarily by lexical factors; due to the limited literature on the acoustic properties of AI-generated speech, these predictions were exploratory.

## 2 Experiment 1

The aim of Experiment 1 was to examine how various factors affect Alexa’s recognition accuracy for words that were produced by human speakers in real time. To accomplish this, native speakers of American English prompted Alexa to spell target words (e.g., “*Alexa, I want you to spell tack*”), and we coded the accuracy of Alexa’s response.

### 2.1 Materials and methods

#### 2.1.1 Participants

All participants (*n* = 21) who served as interlocutors for Alexa were native speakers of American English with no known speech or language impediments. Participants were recruited through campus advertisements and announcements made in classes at the University of Wisconsin-Milwaukee. Fourteen participants were female, six were male, and one participant preferred not to disclose their gender identity; their mean age was 23 years (range: 18–44).

Although dialect wasn’t an exclusionary criterion for the present study, all participants indicated they use the North dialect (Labov et al., 2006). The North dialect is a variation of American English that is used primarily in Wisconsin, Illinois, Minnesota, and Michigan. Three participants indicated that they grew up in bilingual households (Spanish), though their dominant language was still American English. Participants were asked to list their estimated proficiency in languages other than English. Fifteen participants out of 21 indicated various proficiencies in their L2, ranging from beginner to advanced, though none of these participants indicated native-like proficiency in that language. In addition, participants were asked about their prior experience using intelligent voice assistants, such as Amazon’s Alexa, Apple’s Siri, and Google’s Google Assistant. The majority of the participants said they had experience with them, with only four saying they had never used any of them.

#### 2.1.2 Stimuli

Target words were 120 CVC English words, evenly divided into four groups: high frequency/high density (HFHD), high frequency/low density (HFLD), low frequency/high density (LFHD), and low frequency/low density (LFLD). The target words contained one of the ten vowels /ɑ, æ, ɛ, i, ɪ, u, ʌ, oʊ, eɪ, ɑɪ/. Vowels were balanced across the four groups to ensure that they are not a confounding factor on Alexa’s recognition of the target word (see the Table A1 for a complete list of stimuli).

Frequency and density statistics were taken from the English Lexicon Project database^1^ (Balota et al., 2007). In the present study, high frequency words were defined as words with log-transformed values (Log_Freq_HAL) greater than 9. High phonological density words were defined as words having densities (Phon_N) greater than 25. The mean log-transformed frequency and the mean density for the four groups are shown in Table 1. The mean log-transformed frequency for high-frequency and low-frequency words was 10.64 (*SD* = 1.03) and 7.05 (*SD* = 1.11), respectively. The mean density for high-density words was 29.4 (*SD* = 4.20), and 14.7 (*SD* = 3.86) for low-density words. Finally, 30 CVC filler words were purposefully chosen to have mean frequency and mean density values that fell in-between those of our target words (see Table 1).

**Table 1:** Characteristics of stimuli.

	Mean log-transformed frequency (SD)	Mean density (SD)
HFHD	10.64 (1.06)	30 (4.62)
HFLD	10.65 (1.01)	14 (4.12)
LFHD	7.00 (0.98)	29 (3.80)
LFLD	7.11 (1.24)	15 (3.64)
Filler	9.24 (1.67)	23 (0.66)

SD represents standard deviation.

#### 2.1.3 Procedure

Participants first completed a short questionnaire, which was designed to examine their language background. They were then invited to a sound-attenuated room, where the actual experiment took place. There were two conditions for each participant: a normal-speaking rate condition, as well as a fast-speaking rate condition. During the normal-rate condition, participants were instructed to speak at the same speed as they would in everyday life. During the fast-rate condition, they were instructed to speak more quickly than they typically would. The order of the conditions was counterbalanced with half of the participants completing the normal-speaking rate condition first, and the other half completing the fast-speaking rate condition first. In each condition, participants were provided with a printed list of words and were asked to produce each word within the frame sentence, “*Alexa, I want you to spell _______*.” Words were recorded in a sentence even though the sentence itself did not provide any context for the target words. This was done because speaking rate manipulation was expected to be more natural for sentences than for single words. The word list contained 150 stimulus words in total (120 targets + 30 fillers). The order of the words was randomized for each participant. Note that the word *Alexa* was the wake word we used for Alexa. In response to the request, Alexa typically responded in the following format: “______ is spelled ______” (e.g., “*Tack is spelled T-A-C-K*”).

In addition to the word list, participants were also provided with a printed set of instructions that described the process for moving from one word to the next. Regardless of whether or not Alexa spelled each word correctly, participants were asked to pronounce each word only once and move onto the next word. There were two exceptions to this. The first exception occurred when participants corrected themselves immediately after realizing they had mispronounced the target word. The second exception occurred when participants repeated themselves immediately after realizing that Alexa was not awake. Although participants were instructed to verify that Alexa responded to the wake word before asking it to spell each word, some participants failed to do so.

During the experiment, participants were seated next to an Alexa device (2^nd^ Generation of Echo Show). The productions were recorded directly onto a desktop computer running Audacity software at a sampling frequency of 44.1 kHz and 32-bit quantization via a Behringer XM8500 cardioid microphone that was located about five inches from the lips and connected to an M-Audio M-Track Solo preamplifier. The entire experiment took roughly 1 hour, and participants were instructed to take breaks as needed. The participants gave informed consent and received either a gift card or extra credit for their participation.

#### 2.1.4 Coding of data

After listening to the recordings, we categorized Alexa’s responses into three groups: correctly recognized, incorrectly recognized, or excluded (see Table 2). When Alexa’s response was the same as the target word (e.g., *tack* → *tack*) or a homophone of the target word (e.g., *pick* → *pic*), the target word was judged to be correctly recognized by Alexa. Because we tried to avoid words with homophones when choosing our stimuli, *pick* turned out to be the only word to which Alexa responded with a homophone.

**Table 2:** Number of tokens analyzed for each category.

	Category	Number
Counted as correct	Alexa’s response was the same as the target word.	3,921
	Alexa’s response was a homophone of the target word.	12
Counted as incorrect	Alexa spelled a different word than the target word.	771
	Alexa responded with a phrase indicating that it did not know the answer.	127
	Alexa produced a beep sound.	38
Excluded	Participant error	11
	Experimenter error	18
	Alexa did not respond.	85
	Alexa gave related information, however it did not spell the target word.	19
	Alexa gave unrelated information.	25
	Alexa responded with a phrase indicating that it was confused.	13
	Total	5,040

In contrast, when Alexa’s response was different from the target word, the target word was judged to be incorrectly recognized by Alexa. There were four specific cases that met this categorization: (1) The first (and most common) case was Alexa spelling a word other than the target word (e.g., *tack* → *tech*). (2) Alexa responded with a phrase indicating that it did not know the answer (e.g., “*Sorry, I do not know that one.*”). (3) Alexa produced a beep sound instead of spelling a word. The reason why we classified cases (2)–(3) as misrecognition errors is because Alexa often gave those answers in response to specific target words across different participants, suggesting that Alexa understood the question but had trouble recognizing the target words. For example, Alexa almost always gave a beep sound in response to the word *moan*, suggesting that the cause of its response is most likely to be the misrecognition of the word.

In addition, there were six specific cases in which we decided to omit the data, rather than marking Alexa’s response as correct or incorrect. These cases also shared a common characteristic, which was that they could be attributed to random error, and as a consequence, were not consistently observed across different participants or target words. We excluded these tokens, as the cause of the misrecognition was unclear. (1) First, we omitted the errors that participants made themselves (e.g., mispronunciations, or skipping of the target word). (2) Experimenter errors were also omitted (for some participants, the word *both* appeared twice on the word list). (3) There were instances in which Alexa simply did not respond. (4) Alexa gave related information to the target word rather than the spelling of the word (e.g., “*The letter f is the first letter in the word fit*,” when the target word is *fit*). (5) Alexa gave information that did not seem to be related to the target word (e.g., “*The Spell is a book by Heather Killough-Walden*,” when the target word is *hoot*; and “*Alarm for what time*?,” when the target word is *sip*). (6) Alexa responded with a phrase indicating that it did not understand the question or that it was confused (e.g., “*Hmm, did not quite catch that. Say that again?*” “*Please provide more context or information in order to help you*”).

The number of words analyzed per participant was 240: 30 words × 2 levels of frequencies (high, low) × 2 levels of phonological neighborhood density (high, low) × 2 types of speaking rate (normal, fast). Ideally, this would give us 5,040 (240 × 21 participants) tokens to analyze. After excluding 171 tokens for the various reasons listed above, the final dataset included 4,869 tokens.

### 2.2 Results

We performed two analyses on the accuracy data using the *glmer* function in the *lme4* package (Bates et al., 2015) in R, version 4.2.3 (R Core Team, 2023). In the first analysis, our mixed-effects logistic regression model included word frequency (high vs. low), phonological neighborhood density (high vs. low), and speaking rates (normal vs. fast) as fixed effects. These categorical variables were sum coded. The random effect structure included by-subject and by-item random intercepts, along with the maximal random slopes that resulted in convergence (Barr et al., 2013; Matuschek et al., 2017; Brauer and Curtin, 2018). The syntax of the final model was: glmer(accuracy ~ frequency*density*rate + (frequency + density + rate|subject) + (rate|item), family = binomial, data = datafile, control = glmerControl(calc.derivs = FALSE)). In addition, we conducted post-hoc pairwise comparisons for significant interactions using the *emmeans* function of the *emmeans* package (Lenth, 2024). All reported *p*-values were Bonferroni corrected for multiple comparisons.

**Figure 1:**
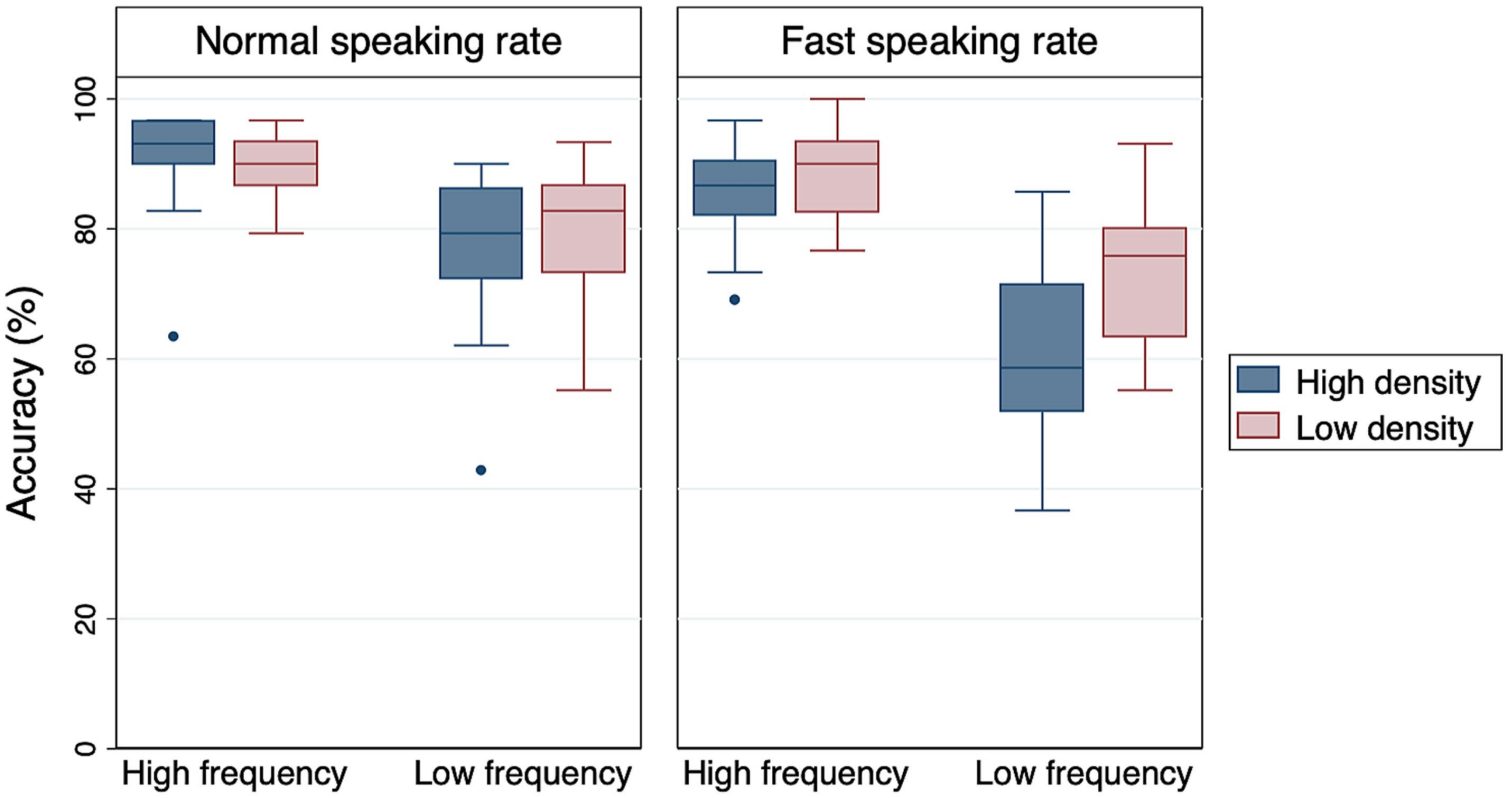
Boxplots of Alexa’s accuracy data. The middle line of each box represents the median value, the outer margins of each box represent the interquartile range, and the whiskers extend to 1.5 times the interquartile range.

As shown in Table 3, the main effects of frequency and rate were significant. High-frequency words were easier for Alexa to recognize than low-frequency words. Also, words spoken at a normal speaking rate were easier for Alexa to recognize than words spoken at a fast speaking rate (Figure 1). Although the main effect of density was not significant, there was a significant density × rate interaction, suggesting that the effect of density was modulated by speaking rate. Post-hoc pairwise comparisons revealed that low-density words were recognized more accurately than high-density words only under the fast rate condition (*Estimate* = −0.963, *SE* = 0.421, *Z* = −2.287, *p* < 0.05). Frequency × density and frequency × rate interactions were not significant, suggesting that the observed difference between high- and low-frequency words held true across different density and rate conditions. Finally, the frequency × density × rate interaction was not significant either.

**Table 3:** Results of mixed-effects logistic regression model.

	Estimate	SE	Z	*p*-value
Intercept	2.695	0.279	9.665	< 0.001
Frequency	0.933	0.227	4.106	< 0.001
Density	−0.285	0.223	−1.274	0.203
Rate	0.513	0.099	5.158	< 0.001
Frequency × Density	0.037	0.222	0.166	0.868
Frequency × Rate	−0.094	0.067	−1.416	0.157
Density × Rate	0.197	0.066	2.978	< 0.01
Frequency × Density × Rate	−0.043	0.066	−0.649	0.517

**Figure 2:**
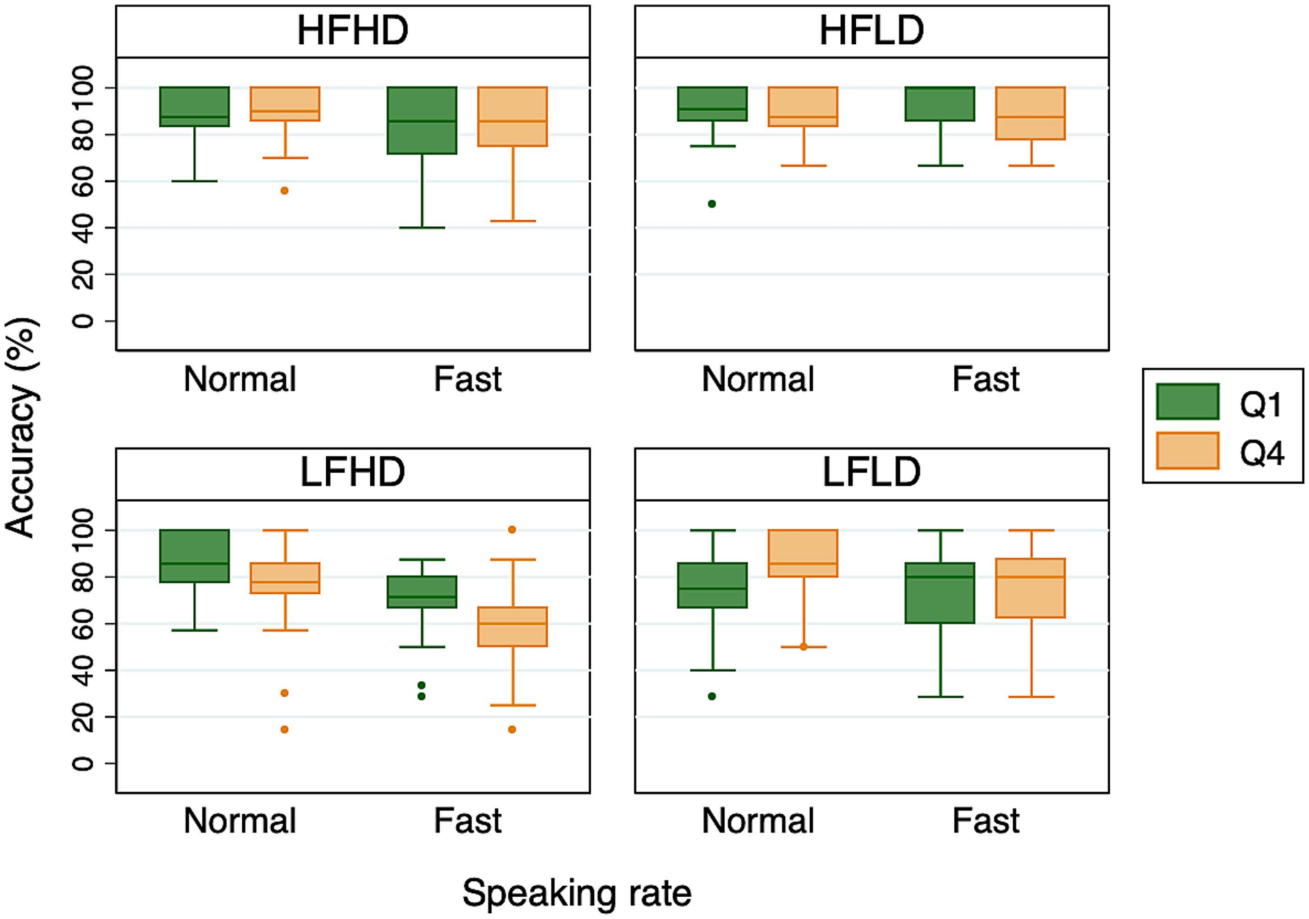
Boxplots of Alexa’s accuracy for the first (Q1) and fourth (Q4) quartiles of the experimental sessions. The middle line of each box represents the median value, the outer margins of each box represent the interquartile range, and the whiskers extend to 1.5 times the interquartile range.

In the second analysis, we examined whether Alexa’s recognition accuracy improved as its experience with the speaker’s voice increased. Specifically, following Bradlow and Pisoni (1999), we looked at the possibility that words presented in the fourth and final quartile (Q4) of the experimental session – that is, later in the session – would be recognized more accurately than words presented in the first quartile (Q1) – that is, earlier in the session. Since each participant produced 120 target words in each speaking rate condition, the first and last 30 target words Alexa heard made up Q1 and Q4, respectively. Figure 2 shows the percent correct accuracy for each of the four word categories presented during Q1 and Q4 of the sessions. We examined the differences between Q1 and Q4 separately for each word category to rule out the possibility that Alexa’s recognition accuracy was impacted by the types of words that made up Q1 and Q4. Each mixed-effects logistic regression model included sum-coded quartile (Q1 vs. Q4) and speaking rate (normal vs. fast) as fixed effects. The random effect structure included by-subject and by-item random intercepts, and maximal random slopes. The syntax of the final model was: glmer(accuracy ~ quartile*rate + (quartile*rate |participant) + (rate|item), family = binomial, data = datafile, control = glmerControl(calc.derivs = FALSE)).

The results indicated that Alexa’s recognition accuracy differed between Q1 and Q4 for two of the word categories: LFHD and LFLD. For LFLD words, Alexa’s recognition accuracy indeed increased from Q1 to Q4, as indicated by a significant effect of quartile (*Estimate* = −0.381, *SE* = 0.169, *z* = −2.253, *p* < 0.05). However, the difference between Q1 and Q4 was driven by the normal speaking rate condition (see Figure 2), as suggested by a significant interaction between quartile and speaking rate (*Estimate* = −0.366, *SE* = 0.176, *z* = −2.083, *p* < 0.05). For LFHD words, which are comparable to the “hard” words in Bradlow and Pisoni (1999), Alexa’s recognition accuracy unexpectedly *decreased* from Q1 to Q4 (*Estimate* = 0.385, *SE* = 0.143, *z* = 2.698, *p* < 0.01). This was observed in both speaking conditions, as indicated by a lack of interaction between quartile and speaking rate (*Estimate* = 0.055, *SE* = 0.140, *z* = 0.390, *p* = 0.696). In addition, we found a significant effect of speaking rate on accuracy for both LFLD and LFHD words. For the other two word categories, HFHD and HFLD, no difference was found between Q1 and Q4, or between normal and fast speaking rates. Thus, overall, we did not find any consistent effect of quartile. This implied that having more experience with the speaker’s voice did not translate into an improvement in Alexa’s recognition accuracy.

### 2.3 Interim summary of Experiment 1

When responding to target words produced by human interlocutors, Alexa was overall more accurate for high-frequency words and for speech produced at a normal rate, compared to low-frequency words and speech produced at a fast rate. Alexa also recognized words with low neighborhood density more accurately than those with high neighborhood density, but only at fast speaking rates. There was no consistent effect of quartile, suggesting that Alexa’s perception performance did not improve over the course of sessions with individual talkers.

## 3 Experiment 2

The aim of Experiment 2 was to examine how various factors affect Amazon Polly’s word production. To accomplish this, we provided carrier sentences to Polly in text form (e.g., “*Alexa, I want you to spell tack*”), which Polly converted to speech. We then analyzed the acoustic characteristics of the target word’s vowel.

### 3.1 Materials and methods

The production experiment used the same 120 target words and 30 filler words as described for the perception study. Each word was embedded in the sentence frame, “Alexa, I want you to spell ______.” The stimulus sentences were generated using Amazon Web Service’s Polly Text-to-Speech tool. We used the standard engine, which was the only engine available in our region. The language was American English, and we used all of the available voice settings, which consisted of five female voices (Salli, Kimberly, Kendra, Joanna, Ivy) and three male voices (Matthew, Justin, Joey), for a total of eight virtual “participants.” for each voice, we generated the full list of 150 sentences twice, once with a normal speaking rate, and once with fast speaking rate. Within each voice and rate, the order of the sentences was randomized.

The rates were controlled using the emphasis level tag in the Speech Synthesis Markup Language (SSML). According to the SSML documentation, the “moderate” emphasis level serves as Polly’s default, so this setting was used for the normal-speaking rate condition. By comparison, the “reduced” emphasis level decreases the volume and speeds up the speaking rate, so this setting was used for the fast-speaking rate condition. Although the changes to volume are not relevant to the current study, we used the emphasis tag because, as far as we are aware, it is the only means of manipulating speaking rate in Polly. The output of Polly was MP3 files, which we converted to WAV files using Praat.

We segmented each WAV file in Praat, using visual inspection of the waveform and spectrogram to indicate the start and end point of each target word and, within each word, the target vowel. We used a script to measure the duration of each vowel, as well as F1 and F2 at the midpoint of the vowel. Fillers were excluded from analysis. Thus, a total of 1920 vowels were analyzed (120 target words × 2 speaking rates × 8 “participants”).

### 3.2 Results

Descriptive results for the production study are displayed in Tables 4–6. We performed statistical analysis on three output variables, duration, F1, and F2, using the *lme* function in the *nlme* package (Pinheiro and Bates, 2000; Pinheiro et al., 2024) in R. Each of the three mixed-effects regression models used the same sum-coded fixed effects as described for the perception study. The random effects structure included by-subject and by-item intercepts; more complex models failed to converge. The syntax of the final model for duration was: lme(duration ~ Frequency*Density*Style, random = list(~1|Participant,~1|Item)). The syntax of the final models for F1 and F2 was the same.

**Table 4:** Mean vowel durations (standard deviations) in seconds, for production experiment.

		Normal rate	Fast rate
High frequency	High density	0.232 (0.059)	0.165 (0.044)
	Low density	0.241 (0.064)	0.173 (0.047)
Low Frequency	High density	0.235 (0.062)	0.169 (0.045)
	Low density	0.237 (0.068)	0.167 (0.049)

**Table 5:** Mean vowel F1 values (standard deviations) in Hertz, for production experiment.

		Normal rate	Fast rate
High Frequency	High density	639.64 (262.03)	639.35 (259.47)
	Low density	626.40 (248.20)	631.57 (249.23)
Low Frequency	High density	644.21 (257.72)	647.15 (259.74)
	Low density	638.57 (256.26)	644.56 (258.94)

**Table 6:** Mean vowel F2 values (standard deviations) in Hertz, for production experiment.

		Normal rate	Fast rate
High Frequency	High density	1813.54 (526.83)	1805.73 (521.52)
	Low density	1851.58 (519.48)	1837.43 (524.73)
Low Frequency	High density	1817.10 (511.17)	1816.66 (516.62)
	Low density	1786.00 (529.56)	1815.92 (512.26)

For duration, there was a significant effect of speaking rate (*Estimate* = −0.034, *SE* = 0.001, *t* = −66.468, *p* < 0.05), such that vowels in the fast condition were significantly shorter than vowels in the normal condition. No other effects or interactions were significant. For F1 and F2, no effects or interactions were significant.

### 3.3 Interim summary for Experiment 2

The results of Experiment 2 showed that the Polly’s target vowels had significantly shorter duration at a fast speaking rate, compared to a normal speaking rate. Rate had no significant effect on vowel formants. Furthermore, lexical characteristics had no significant effect on any acoustic measurement.

## 4 Discussion

The current study examined how Amazon Alexa and Polly responded to lexical and stylistic factors that are known to modulate speech perception and production in humans. In the domain of perception, Alexa’s performance was significantly affected by word frequency and speaking rate. Its performance was also affected by neighborhood density, but only at fast speaking rates. Alexa showed no evidence of adaptation over time; although its performance changed from the first quartile of target words (Q1) to the fourth and final quartile (Q4) for low frequency words, the change was in an unexpected direction. Meanwhile, in the domain of production, Polly’s performance was significantly affected by speaking rate alone. Overall, these findings suggest that AI speech lacks some of the flexibility that is the hallmark of human speech. In what follows, we discuss each of these findings in turn.

### 4.1 Perception and frequency

Alexa’s recognition performance was significantly affected by frequency, such that it exhibited higher accuracy for frequent target words, compared to infrequent ones. This result is almost certainly expected. Although the precise algorithms underlying AI models are proprietary, the key idea of such models is that they produce output using the occurrence of already-existing patterns within very large datasets. It stands to reason, then, that AI models would make a basic distinction between frequent versus infrequent patterns.

With that said, our results are still notable because frequency exerted an impact on accuracy scores even under “ideal listening” conditions – that is, in a quiet environment with no time pressure for a response. In comparable conditions, human listeners performing lexical decision tasks (a common measure of word recognition) typically produce accuracy scores which are at ceiling (Luce and Pisoni, 1998); the difference between frequent versus infrequent words surfaces primarily under conditions of pressure, or through finer-grained measures such as reaction time. That is, even if a human takes a little bit longer to respond to low-frequency words like *gush*, he or she will ultimately respond to them with high accuracy. Such is not the case with Alexa, whose basic ability to recognize low-frequency words is compromised. In this regard, Alexa’s behavioral response to frequency is parallel to—and yet more extreme than—a human listener.

Also, it is worth mentioning that there might be differences between the words that human listeners and AI models find frequent. For instance, although the word *dune* was one of the low-frequency words according to the database we used, Alexa recognized the word with a startlingly high accuracy rate of 97.6%. Given that *Dune* is the title of a widely-seen film, we suspect that it was one of the frequent words in the datasets Alexa was trained on.

### 4.2 Perception and density

Alexa’s overall recognition performance was not significantly affected by density. This differs from human listeners, who typically recognize low-density words better than high-density ones. For humans, this effect is attributed to a process of lexical competition. Specifically, an input signal will activate not only its target, but also any stored lexical representations which sound sufficiently similar to the target. When a larger number of representations are activated (as is the case for high-density words), there are more potential words competing for recognition, so perception becomes less accurate and slower. In our results, the lack of a density effect could conceivably suggest that Alexa simply does not use a competition algorithm, or that if it does, the competition does not involve similar-sounding words.

Such a conclusion does not seem entirely warranted, however, because our results do show an effect of density at fast speaking rates. In this condition, where speech segments are more rapid and more reduced, high-density words exhibited lower accuracy rates than low-density words. This finding seems to suggest that Alexa does employ a competition algorithm, at least in some conditions, or alternatively that Alexa is sensitive to a very closely related measure, such as the transitional probability between phonemes.

Despite the absence of a main effect for density, this interaction effect is consistent with what we know about human perception more broadly—namely, that certain effects which are dormant under ideal listening conditions will manifest themselves under more difficult conditions or, as Bradlow and Pisoni (1999, p. 2075) put it, “perceptual difficulties introduced by one factor might be attenuated or amplified by the presence of another factor.”

### 4.3 Perception and speaking rate

Alexa’s recognition performance was affected by speaking rate, such that accuracy was lower at the faster rate. This is comparable to findings that have been reported for human listeners. This finding is particularly important in light of recent studies on the acoustic properties of Alexa-directed speech, which have shown that human speakers slow down when speaking to Alexa (Cohn and Zellou, 2021; Cohn et al., 2021). It is assumed in these studies that human speakers adjust their speaking rate in order to ensure that Alexa recognize the word. Nevertheless, there has not been any empirical evidence to suggest that the speaking rate adjustments will actually enhance Alexa’s word recognition. By demonstrating that Alexa’s word recognition rate was significantly impacted by the speaking rate of the sentences that were produced “live” by human speakers, our study provides a basis for understanding the speaking rate modifications found in the previous research.

In order to estimate the size of the acoustic difference between the normal and fast speaking rates used by Alexa’s human interlocutors (Experiment 1) as well as Polly (Experiment 2), we measured speaking rate for a subset of the utterances that they produced. Specifically, we calculated the speaking rate of the frame sentence “I want you to spell ____” that contained the following four words: *seat* (HFHD), *check* (HFLD), *hag* (LFHD), and *robe* (LFLD). The four words were chosen because in Experiment 1, they exhibited the recognition accuracy closest to the median accuracy in each word category. In total, 168 sentences (4 sentences × 2 speaking rates × 21 participants) were analyzed for Experiment 1, and 64 sentences (4 sentences × 2 speaking rates × 8 voices) were analyzed for Experiment 2.

The results showed that the human speakers in Experiment 1 produced an average of 4.37 syllables per second (SD = 0.71) under the normal speaking rate condition, and 6.51 syllables per second (SD = 1.19) under the fast speaking rate condition. In Experiment 2, Polly produced an average of 3.50 syllables per second (SD = 0.34) under the normal speaking rate condition, and 4.90 syllables for second (SD = 0.44) under the fast rate condition. These results help us verify that the speakers in both experiments indeed produced the rate differences that we expected. Interestingly, Polly exhibited a smaller difference between normal and fast rates compared to human speakers, as well as a slower rate overall.

### 4.4 Perception and adaptation over time

Alexa’s recognition performance changed from Q1 to Q4, but this effect was only found for low-frequency words. To some extent, this is not surprising, because Alexa’s performance was near ceiling for high-frequency words (see Figure 1). When looking at results for low-frequency words, however, we do not necessarily see the trajectory of change that we would expect. In our results, recognition accuracy for LFLD words did increase from Q1 to Q4, but this effect was limited to the normal-rate condition. This is not what we see in previous results from human listeners: Bradlow and Pisoni (1999) demonstrated improved perception over time, but their effect was limited to the fast-rate condition (their study examined “difficult” words, namely LFHD words), where recognition was presumably more difficult to begin with. Puzzlingly, Alexa seems to exhibit improvement in a condition where none is needed.

Also in our results, recognition accuracy for LFHD words actually *decreased* from Q1 to Q4. This finding, which held across participants and items, directly contradicts what we expect from adaptation, and does not have a clear explanation. It is possible that our human speakers experienced fatigue, and therefore devoted less and less effort to their pronunciations as they progressed through the experiment, thereby producing less recognizable words in Q4 compared to Q1. However, it is unclear why fatigue should impact only one category of words (LFHD) to the exclusion of the others.

In sum, although we found some evidence of change over time, we cannot characterize these changes as adaptation. Further work is needed to understand how and why Alexa’s accuracy rates fluctuate as it interacts with an individual speaker. For now, we can conclude that Alexa lacks the perceptual flexibility that is a hallmark of human speech perception.

### 4.5 Comparison with previous studies of human perception

Using many of the same variables employed in the current study, Bradlow and Pisoni (1999) examined word recognition in human listeners. Table 7 displays their accuracy results and compares them to ours. Note that although the current study used four types of words (2 levels of frequency × 2 levels of density), the comparison depicts only two types of words (“easy” versus “hard,” corresponding to HFLD versus LFHD), since those were the two types examined by Bradlow and Pisoni (1999).

**Table 7:** Recognition accuracy for the easy and hard words gathered from human listeners in Bradlow and Pisoni (1999) and from Alexa in the current study.

	Speaking rate	Bradlow and Pisoni (1999)	Current study
Easy word (≈HFLD)	Medium	94.67%	89.69%
	Fast	91.61%	89.07%
Hard word (≈LFHD)	Medium	90.04%	77.52%
	Fast	84.97%	59.76%

Several patterns are apparent. First, Alexa’s accuracy rate is lower than that of humans overall. Although Alexa performs well enough to be used as an everyday commercial product, its basic capacity for word recognition still falls short. This may be due, in part, to the nature of Alexa’s training data, which presumably consists of words occurring in meaningful contexts. By contrast, the stimulus sentences for the current study (“*Alexa, I want you to spell ____*”) were stripped of context, thereby depriving Alexa of one of its most valuable clues.

Second, Alexa’s accuracy is more strongly affected by a change from easy to hard. That is, while human recognition rates are roughly comparable for easy and hard words, Alexa’s recognition rates are noticeably lower for hard words. With the caveat that hard words are actually a composite of both low frequency and high density, it appears that Alexa is overly sensitive to frequency effects. Given the basic premise of AI models, this is perhaps not surprising, and it highlights the need for such models to integrate additional principles, besides frequency alone.

Third, Alexa and human listeners are affected by speech-rate changes in different ways. For easy words, Alexa’s accuracy is *less* affected by changes in rate. This suggests that Alexa may have already been trained on fast speech samples, and indeed, that it may have had relatively more exposure to fast speech than humans have. But for hard words, Alexa’s accuracy is *more* affected by changes in rate. Thus, although Alexa has a basic capacity to respond to fast speech, this capacity deteriorates with low-frequency, high density words, providing further support to the notion that Alexa is oversensitive to frequency.

Although we have sketched a basic comparison of perception in humans versus Alexa, the conclusions that we can draw from Table 7 remain limited by differences in stimulus design and experimental procedure. Future work could elaborate the comparison by directly comparing humans and Alexa as they respond to the exact same stimuli and with the exact same procedure.

### 4.6 Production

In contrast to Alexa’s perception performance, which mimicked some of the capacities of human listeners, Polly’s production performance suggests only a very limited approximation of human speakers. The results did show that the duration of vowels in the target words varied as a function of speaking rate, with shorter durations in the fast rate. However, such a result would be expected under any definition of “rate.” Meanwhile, vowel quality (as measured by F1 and F2) did not vary as a function of speaking rate. This differs from what has been typically reported for human speakers, who produce expanded vowel spaces in clear speech, which is typically spoken at a slower rate, compared to casual speech, which is typically spoken at a faster rate (although we note that speech style and speech rate, while closely linked, are nevertheless distinct concepts). Thus, whereas humans seem to accomplish changes in speech rate by harnessing a variety of linguistic variables, Polly limits itself to non-spectral changes.

Furthermore, results provided no indication that Polly’s productions were modulated by lexical characteristics. This clearly differs from what has been reported for humans. In human speech, high-frequency words have shorter durations and more contracted vowel spaces than low-frequency words. Interpreted within the framework of Lindblom’s H&H theory, this is because human speakers know that frequent words are easier for human listeners to recognize, and they can therefore devote less effort to their articulation. Essentially, this is a “theory of mind” scenario, in which the speaker attempts to understand the mental state of the listener, and acts accordingly. If this scenario is correct, then the lack of a frequency effect for Polly’s productions need not be a surprise, because Polly is an AI model that lacks theory of mind.

For density, the previous research on human speech is mixed. As noted in the Introduction, some studies have reported that humans produce high-density words more clearly. This scenario can be interpreted within the framework of H&H theory in the same manner as frequency; that is, because human speakers know that high-density words are more difficult for human listeners to recognize, they produce them with longer durations and more expanded vowel spaces. However, other studies have reported that humans produce high-density words *less* clearly, possibly because they devote less effort to sequences of sounds that are highly practiced. Although these notions are quite different from one another – H&H’s theory of mind, on the one hand, and the concept of “effort,” on the other—neither of them is applicable to a software algorithm. Interpreted in this way, Polly’s lack of sensitivity to lexical characteristics is not entirely surprising.

With that said, the ultimate test of Polly’s speech production would not really come from acoustic measurements such as those that we report here (which are themselves limited, since we examined only the vowel in CVC target words). Instead, it would come from human listeners themselves, who could provide data to help us determine whether the lack of frequency- and density-based acoustic modulation affects their ability to recognize words. Future perceptual studies could use Polly-generated speech to accomplish this goal.

The current study used the standard engine of the AWS Polly Text-to-Speech tool, which was the only engine available in the region where this research was conducted. Other regions have access to the neural engine, and its output could potentially be quite different from what we examined here. The current study is also limited to speech output produced by a single commercial organization; the output produced by other commercial organizations, or by research groups, could potentially be quite different.

### 4.7 Future work

For future work on AI tools, a crucial question will concern dialect variation. While the current study has examined both lexical and stylistic sources of variation, and also touched on questions of indexical variation, it has done so within a relatively homogenous dialectal context. For the perception study, our human speakers were native speakers of a variety of American English that is associated with white people living in the midwestern region of the United States. For the production study, the voices supplied by Polly possessed similar characteristics. But much of the richness of human language – and much of the variation in the speech stream – comes from dialects. Understanding how AI tools respond to dialectal variation is important for basic linguistics and cognitive science, because we know that humans adapt very rapidly to features of a novel dialect, both in perception (e.g., Dahan et al., 2008) and in production (e.g., Babel, 2010, 2012). Thus, in order to have a full scientific understanding of tools like Alexa and Polly, we must characterize the extent to which they adapt, or fail to adapt. Work on dialectal variation is also important for ethical reasons, because previous research has demonstrated that AI tools exhibit biases to dialects. For example, language models have been shown to exhibit racial disparities in their responses to African-American speech (Koenecke et al., 2021) and also to embody less overt racism in the form of prejudice against African-American dialects (Hofmann et al., 2024). Future work must continue to probe these issues.

## 5 Conclusion

AI tools seem to mimic human speech in ways that were unimaginable until very recently. But how good is their mimicry? The answer to this question is important because it will affect our own human behavior when we interact with AI. The current study brought two AI tools, Alexa and Polly, “into the lab” and tested them with some of the same variables that have been shown to shape human perception and production: frequency, density, speaking rate, and change over time. Our results showed that for some of these variables, AI tools exhibited sensitivity in the same manner as humans; for others, however, AI tools exhibited oversensitivity, or no sensitivity at all. These findings deepen our understanding of what artificial intelligence can – and cannot – do.

## Data Availability

The raw data supporting the conclusions of this article will be made available by the authors, without undue reservation.

## Footnotes

^1^ http://elexicon.wustl.edu

## Appendix

**Table A1:** Stimuli used in Experiments 1 and 2.

	Vowel	HFHD	HFLD	LFHD	LFLD
1	ɑ	pot	top	cot	mop
2	æ	sad	van	lad	sag
3	æ	fat	half	sap	dash
4	æ	pack	gas	pap	gaff
5	æ	pat	path	tack	zap
6	æ	bad	jazz	hag	dab
7	ɛ	bet	death	peck	pep
8	ɛ	set	check	beck	chef
9	i	seek	teeth	beak	beef
10	i	feed	league	bead	deem
11	i	sheet	deep	beep	sheath
12	i	seat	teach	seep	jeep
13	ɪ	bit	zip	sip	ditch
14	ɪ	tip	kiss	hick	miff
15	ɪ	pick	fish	wick	hiss
16	ɪ	bid	give	hid	fig
17	ɪ	fit	ship	pip	pith
18	u	suit	youth	hoot	goose
19	u	moon	food	dune	womb
20	u	boot	duke	toot	booth
21	ʌ	cut	such	hut	shuck
22	ʌ	bug	judge	bun	gum
23	ʌ	suck	tough	putt	gush
24	oʊ	note	both	soak	dose
25	oʊ	bone	home	moan	robe
26	eɪ	gain	gave	fade	babe
27	eɪ	hate	tape	bake	nape
28	eɪ	date	shape	sate	Jake
29	ɑɪ	like	pipe	kite	hike
30	ɑɪ	fight	wife	tyke	wipe
